# Success of Xenografts in Alveolar Ridge Preservation Based on Histomorphometric Outcomes

**DOI:** 10.3390/dj11090215

**Published:** 2023-09-13

**Authors:** Rabia S. Khan, Mohsin Aslam, Cemal Ucer, Simon Wright

**Affiliations:** 1 Department of Material Science and Engineering, Faculty of Science of Technology, University of Lancaster, Lancaster LA1 4YR, UK; 2 ICE Postgraduate Dental Institute and Hospital, University of Salford, 24 Furness Quay, Salford M50 3XZ, UK; drmohsinaslam@outlook.com (M.A.); cemalucer@me.com (C.U.); profwright@glencairndental.co.uk (S.W.)

**Keywords:** alveolar ridge preservation, biomaterials, histomorphometry, xenograft

## Abstract

Different xenograft approaches in alveolar ridge preservation (ARP) are essential to understand relative to their histomorphometric outcomes. Therefore, the aim of this study involved studying biomaterials of a xenograft nature that are used in ARP procedures, to compare the different approaches and evaluate their efficacy in relation to histomorphometric data. An electronic search was completed using the databases: Ovid (Medline), Google Scholar and Wiley Online Library, including a hand search for relevant articles and grey literature. Only randomised controlled trials, using xenograft biomaterials for alveolar ridge preservation procedures involving human studies, dated from 2010–2022 were included in the review. An initial search yielded 4918 articles, after application of the eligibility criteria, 18 studies were deemed eligible for inclusion in the systematic review. The two main xenograft groups found were of bovine origin and of porcine origin. The main histomorphometric outcomes evaluated included new bone percentage (N.B%) and residual graft percentage (R.G%). The mean N.B% for the bovine and porcine groups were 33.46% and 39.63% respectively and the mean R.G% for the bovine and porcine groups were 19.40% and 18.63% respectively. The current evidence suggests that the two main xenograft biomaterials used in ARP procedures after tooth extraction, which are of bovine and porcine origin, displayed effectiveness in producing new bone.

## 1. Introduction

Morphological alterations subsequent to tooth extraction can produce a pronounced reduction of bone. This forms a narrow ridge which can account for a reduction of up to 50% of the original alveolar ridge width and a lesser vertical reduction, forming a shorter ridge [1]. The alveolar crest tends to shift to alingual position, two-thirds from the buccal position [2]. A decrease in the alveolar ridge volume after tooth extraction can have a detrimental effect on successful implant placement in relation to the functioning and aesthetics of the prosthesis due to a reduction in the hard and soft tissue architecture. Accordingly, alveolar ridge preservation (ARP) is a procedure done after tooth extraction to reduce these morphological alterations. It preserves the stability of the ridge volume to improve the aesthetic and functional results and simplifies successive treatment procedures [3,4].

Implant treatment should have a prosthodontically focused approach; hence the consideration and use of ARP should be an essential component of implant treatment planning to minimize this reduction in the alveolar ridge subsequent to tooth extraction and provide the best prosthodontic outcome [5]. Furthermore, it has been shown that implants placed in bone grafted areas have a comparable survival rate to that of none grafted bone sites [6].

ARP can be technique-sensitive, and its outcome could be difficult to predict, with some resorption of the alveolar ridge being unavoidable [7]. Consequently, it is imperative that testing of the biomaterials is undertaken to understand which approaches produce superior results so that they can be replicated to produce the best outcomes in clinical practice. Grafting biomaterials are utilized to counteract alveolar bone loss after tooth extraction and can be categorised into assorted groups including autogenous, allografts, xenografts, alloplasts, and platelet concentrates [8]. For many years the gold standard for bone regeneration has been autogenous bone despite the difficulty in harvest, clinical time, and an increase in morbidity [9,10,11]. Alternative biomaterials such as xenografts are more readily accessible, require less clinical time and there is no patient morbidity.

Alternative biomaterials such as xenografts are more readily accessible, require less clinical time and there is no patient morbidity. Xenografts are osteoconductive in nature and acquired from a species that is different from the recipient, they are mainly made from the inorganic part of bone tissue from animals [12].

### Aims and Objectives

The scope of this systematic review will focus on the use of xenografts of bovine and porcine origin in ARP. Considerable previous research has been done regarding the volumetric analysis of the alveolar ridge following ARP, however, significantly less has been done concerning the biology [3]. Hence, the scope of this systematic review is based on the biological parameter of histomorphometry and aims to provide more of an understanding of whether one type of xenograft is superior to another in producing new bone regeneration during ARP. Vital information that would improve outcomes of ARP procedures include how much bone is formed, which biomaterials resorb the most, and which have the least residual graft material after healing.

The primary objective relative to the intervention (ARP) will focus on the histomorphometric outcomes of xenograft biomaterial of bovine and porcine origin only. The objectives in relation to the outcomes are focused on the histomorphometric changes that occur within the alveolar ridge preserved sites in terms of new bone formation and residual graft material to see which xenografts produce the best results. 

Due to the diverse nature of the intervention, secondary objectives will be to identify the different treatment approaches involving xenografts in ARP i.e., the use of a membrane and whether these approaches may affect the outcome of ARP. Furthermore, to improve the evidence which is deficient in previous literature relating to a biological parameter of ARP with the latest literature, and aid in addressing the doubt of the efficacy of biomaterials in relation to their histomorphometric outcomes. 

## 2. Materials and Methods

A robust evidence system that produces a repeatable process of methodically examining data from numerous studies related to one another to form reliable conclusions at the same time as restricting bias and inaccuracies. The PRISMA (Preferred Reporting Items for Systematic Reviews and Meta-Analyses) statement will be implemented which will assist in including matters considered essential for a systematic review for clarity and comprehensive reporting [13]. Prior to the commencement of systematic review, the protocol was not registered.

It was aimed at understanding the different xenografts used in ARP procedures.

How do the different xenografts differ when compared to each other both inter-classification (i.e., porcine vs bovine) and intra-classification (i.e., bovine vs. bovine) with regards to their histomorphometric outcomes.

What are the different interventions involved in ARP procedures using xenografts and do they influence the histomorphometric outcomes.

The study characteristics with respect to the research questions are developed through the PICOS design [14].

### 2.1. Population

This included all patients in studies who meet the respective eligibility criteria and are undergoing a tooth extraction(s) followed by an ARP procedure with a xenograft. It also included participants that are undergoing tooth extraction(s) and not receiving an ARP procedure with a xenograft, which is used as a control to compare the effect of ARP with spontaneous healing. The patients not receiving xenograft may still have other forms of treatment (e.g., membrane use) as part of the study intervention.

### 2.2. Intervention

The intervention consists of a biomaterial of xenograft nature that is placed into the extraction socket(s) after tooth extraction. It also entails participants who are not receiving any biomaterial within the extraction socket(s), although are receiving some other form of post extraction treatment (e.g., use of a membrane) as part of the study intervention.

### 2.3. Comparison

The study primarily compared the effectiveness of different xenografts of bovine and porcine origin, and their various approaches in ARP in relation to histomorphometric outcomes. It will also be comparing the different types of xenografts within each of their respective biomaterials i.e., different bovine xenografts.

Secondarily, due to the different approaches involved in ARP, an indirect comparison of the different protocols utilized in ARP procedures has been undertaken e.g. the influence of primary soft tissue closure versus open healing by secondary intention. Due to the diverse nature of the study characteristics, other factors such as socket morphology, healing time, and apical to coronal differences within the socket following ARP in relation to their histomorphometric outcomes have also been evaluated.

### 2.4. Outcome Measures

The histomorphometric outcomes in terms of new bone formation, connective tissue, and residual graft material percentages will be measured. This will be done following a healing period after the ARP procedure and a subsequent biopsy for histological analysis has been completed, where the constituents of bone from the grafted and non-grafted sites can be compared. Due to the potential diversity of the studies, other outcome measures that do not encompass the headings mentioned above will also be measured if the study utilizes a certain outcome measure e.g., bone area fraction.

### 2.5. Search Strategy

Using the databases: Ovid (Medline), Google Scholar, and Wiley Online Library, an electronic search was completed.

The following key words and MeSH (Medical Subject Headings) terms were used for the Ovid (Medline search) using the resource: Ovid MEDLINE(R) and Epub Ahead of Print, In-Process, In-Data-Review & Other Non-Indexed Citations Daily and Versions(R) 1946 to 24 November 2022-“Histological”, “Histological Techniques”, “Alveolar Ridge Preservation”, “Alveolar Ridge Augmentation”, “Xenografts”, “Heterografts”. The Advanced search tab was used, and the MeSh terms exploded so that the search encompassed references indexed to the subject heading, also including references indexed to any narrower subject headings. Boolean Operators (AND/OR) were used to combine the MeSh terms. A “Publication Types” limit for randomised controlled trial and a limit for “Publication Year” of 2010 to Current, were added to produce a more focused search.

For the Wiley Online Library database, the advanced search tab was selected and the MeSh terms “Xenograft”, “Alveolar Ridge Preservation”, “Histology” was used, no particular field was entered such as Title or Author next to the MeSh terms to keep the search broad. Following this, a “Publication Type” filter of journals, and “Subject” filter of DENTISTRY, and a “Publication Date” of 2010–2022 were applied for a more focused search.

The MeSh terms “Histology”, “Alveolar Ridge Preservation”, “Xenografts”, and “Randomised Controlled Trial” were used in Google Scholar in the advanced search option (with “all the words” option selected “anywhere in the article”). The Boolean Operators (AND/OR) were used to combine the MeSh terms, a customized time filter of 2010–2022 was placed and only articles in English were filtered to narrow the search.

The eligibility criteria were applied whilst conducting a primary screening of the study titles and abstracts for each of the three database searches and hand-searched articles, and potential included studies were selected. A detailed evaluation of the full text of the articles selected from the primary screening was then undertaken to select the final included articles by one examiner (A), and the eligibility criteria were applied to the studies to select the final articles.

### 2.6. Eligibility Criteria

Inclusion Criteria

- Randomised controlled trials (RCTs)
- Publications with patients reporting no systemic disease, with a status of good health.
- Patients with systemic disease may contribute towards poor results due to their poor healing which can lead to skewed and inaccurate results.
- Publications utilising xenograft biomaterials for ARP or studies utilising other biomaterials (x) i.e., allografts, if xenografts are being compared with (x) and against natural healing.
- Publications including the histomorphometric outcomes of xenografts in ARP.
- Publications of a human sample
- Publications from January 2010 to October 2022.

Exclusion criteria

- Studies with <10 participants
- Follow-up periods < 3 months
- Studies that do not take into account the health of patients where participants with systemic disease can be included in the study.
- Studies not observing histomorphometric outcome measures.
- Studies involving different biomaterials other than xenografts and/or studies comparing xenografts and other biomaterials alone, without a control group of natural healing (e.g., comparing xenografts and allografts with no control group of natural healing).

## 3. Results

### 3.1. Study Selection and Search Results 

**Figure 1:**
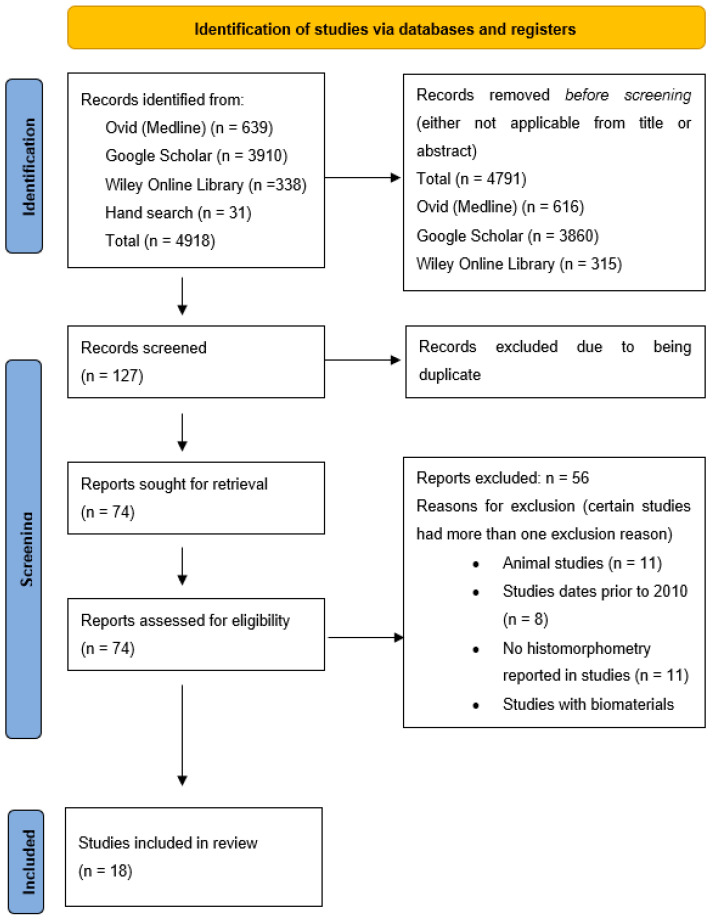
PRISMA flowchart.

An initial search yielded 4918 articles. A total of 639 articles from Ovid (Medline), 3910 articles from Google Scholar, 338 articles from Wiley Online Library, and 31 articles from hand searching. After screening the article titles and abstracts, a total of 4791 articles were removed as they were not applicable to the research question and/or didn’t satisfy the requirements of the eligibility criteria, which left 127 articles for screening see Figure 1. A further 53 articles were excluded due to being duplicates, which left 74 articles for review. After full-text evaluation and applying the eligibility criteria, 56 articles were excluded, which left a total of 18 articles for inclusion for the study. The excluded studies main reasons included animal studies, studies dated before 2010, studies other than RCTs, no histomorphometry reported, and studies reporting biomaterials other than xenografts which had no natural healing group.

### 3.2. Study Design, Population, and Characteristics 

The diversity of the study design, population, and characteristics of the studies in relation to their materials, number of patients and biomaterials all varied significantly. The array of study characteristics, population, and design are outlined in Table 1. The aspects reported in the table are the design of the study, the setting, and the number of centers. Also reported are the mean age, gender, number of sockets in the study and the groups i.e., control and test groups, their relative biomaterials, and confounding factors.

In the 18 included articles, all of them were randomized controlled trials in adherence to the inclusion criteria. All 18 studies had a parallel design, four studies had a split mouth design [15,17,18,27], three studies had a three-arm design [16,20,21], one study had a four-arm design [22]. The age ranged from 20–82. The sample of patients in the studies ranged from 10–90 (mean-31.3) and the number of sockets ranged from 18–90 (mean–37.6).

### 3.3. Intervention Characteristics

The intervention characteristics can be divided into two main aspects 

Interventions involved with extraction and interventions concerned with alveolar ridge preservation (outlined in Table 2). The characteristics reported in Table 2 include the groups i.e., control and test groups, their relative biomaterials, and the number of patients per group. The clinician and skill level who conducted the ARP procedures is reported, as the use of a flapped or flapless extraction, any use of releasing incisions, and whether primary closure was obtained. Additionally, the reasons for tooth extraction, whether the use of an atraumatic technique is implemented, the number and type of teeth/their location, if the socket morphology after extraction is reported, and whether this is taken into consideration in the eligibility criteria.

The characteristics reported in Table 2 are the type of barrier membrane, including the pharmacological aspects including the use of antibiotic prophylaxis, topical rinses, and any medicaments used. Furthermore, whether a temporary prosthesis is present and adjusted, the healing time before biopsy, whether there is any monitoring after ARP, the number of dropouts, any adverse events, and the follow-up times are also reported.

The broad categories of xenografts used were of bovine origin, porcine origin, and enamel matrix derivative (EMD) which are also of porcine origin with regard to the types of materials being investigated. Allografts were included in two studies as xenografts and natural healing were also being tested [28,30], also Plasma Rich Growth Factors were included in one study for the same reason [30]. Bovine biomaterials were the most reported studies, of which Bio-Oss was the most studied biomaterial in nine studies [5,12,20,25,27,28,29,30]. Bio-Oss collagen was the second most studied reported in five studies Alkan et al., [15], Ben Amara et al. [19], Heberer et al. [24] (Mercado et al., 2021), (Nart et al., 2017)) [26,27]. Endobon, a deproteinised bovine bone mineral was reported in two studies [17,29]. The other studies that used bovine bone included Cerabone, a natural bovine bone mineral [30]; MinerOss X, a deproteinised bovine bone mineral Guarnieri, Stefano, et al., [22] and Osseus, another deproteinised bovine bone mineral Calasans-Maia et al., [12]. Bovine biomaterials were studied against bovine bone in six studies [5,12,17,26,27,29]. Bovine biomaterials were studied against natural healing in five studies [19,20,24,28,30]. Bovine against porcine biomaterials were present within two studies [22,25], bovine against EMD in one study [15] (Alkan et al., 2013) and as an adjunct with EMD in another study [26].

Porcine biomaterials were the second most reported studies which included various types of porcine xenograft. Corticocancellous porcine bone was used in three studies (two studies used MP3, one study used Tecnoss. Cortical porcine bone is reported in one study (Apatos), porcine bone mineral (MinerOss XP) is reported in two studies); and one study used a cancellous porcine xenograft (Zcore). Porcine xenografts were studied between one another in two studies ((Antonio Barone et al., 2015), (Antonio Barone et al., 2017) [16,18]; against bovine xenografts [22,25] and against natural healing in three studies [18,23,25]. EMD was the third most prevalent xenograft included in two studies and was studied against Bio-Oss [15] and as an adjunct to Bio-Oss collagen against Bio-Oss collagen [26].

Eleven studies reported a flapless extraction compared to five studies which reported a flapped extraction and one study compared flapped versus flapless extraction. Fourteen studies reported no use of relieving incisions whereas four studies did use a relieving incision. Five studies reported primary closure. One study didn’t report anything on releasing incisions, primary closure or whether a flapped versus flapless extraction was conducted and two studies had no report of an atraumatic extraction. Ten of the studies reported the reasons why the teeth were being extracted which included, caries, periodontal reasons, endodontic failure, fracture, and prosthetic failure. In fourteen studies, the socket defects after extraction were taken into consideration in the eligibility criteria and the tooth types were also taken into consideration in seventeen studies.

Sixteen studies reported the use of a membrane, with the most reported membrane being a collagen membrane in five studies, followed by Bio-Gide (a porcine derived native collagen membrane) in four studies. A collagen sheet (Condress) was used in one study, and two studies reported a porcine derived resorbable collagen membrane (Mem-Lok). Two studies reported an autogenous membrane, one of these studies used a mucosal punch graft combined with a free gingival graft and the other study used a free gingival graft. A further two studies reported synthetic membranes: one study used a dense polytetrafluoroethylene (d-PTFE) membrane, and the other study used a synthetic polymeric resorbable membrane (PEG).

The pharmacological treatment differed vastly in terms of pre-operative and post-operative medications and their duration of use. This included the use of antibiotics, mouthwashes, analgesics, nonsteroidal anti-inflammatory medication, and the application of topical medicaments. Two studies didn’t report any pre-operative or post-operative treatment [17,24]; one study reported pre-operative and post-operative treatments for both the extraction, ARP and the implant surgery [26]. Regarding any temporary prostheses that participants had within the studies, only four studies reported restrictions of use or alterations to make the prostheses free from the surgical site [16,20,28,30]. Three of the studies didn’t report a review after the ARP procedure [16,18,21]; and two of the studies reported complications after the ARP procedure which included grafts becoming necrotic, site opening, membrane exposure and spillage of the graft material [15,17]. The healing times within the studies (from ARP to biopsy) ranged from 3–9 months; and the follow-up times ranged from 12 months–5 years.

### 3.4. Histomorphometric Analysis

The histomorphometric analysis has various components associated with its evaluation which varied considerably. The characteristics reported in Table 3 outline the histomorphometric analysis and include whether the outcome definitions are reported, the method of collecting the biopsy and the dimensional biopsy bone core characteristics. Moreover, the histological treatment of the biopsy, the biopsy location, whether any method was used to obtain a location, and the number of slides analysed are reported. Also reported are the histomorphometric analyses, the granule size of the biomaterials and whether the studies investigated the apical to coronal differences within the biopsies.

### 3.5. Quality Assessment and Risk of Bias 

Examining the studies to evaluate their validity and relevance is an important component of a systematic review. The quality assessment of the studies is outlined in Table 4, they report upon numerous factors including whether the eligibility criteria are defined and whether the study groups are representative of the population. Other aspects reported upon include whether a random sequence is generated, the allocation concealment, if blinding of the participant and examiner is present, if blinding of statistical analyses took place, and if blinding of histological analyses are evident. Additionally, whether there is calibration (both intra and inter-examiner), whether the treatment was identical except for the intervention, reporting of incomplete outcome data and reporting of loss to follow-up are included. Moreover, if selective reporting is evident, the study design, sources of funding, whether informed consent and if ethical approval is implemented are also reported.

The eligibility criteria were present in all eighteen studies. All the studies were RCT’s, and all had a random sequence generation (this varied from computer generation, the envelope method, and a coin toss). Allocation concealment, where the person randomizing the patient doesn’t know what treatment group the participants are allocated to, was not reported in any of the studies. Four studies reported on whether the participants or the examiner (person conducting the ARP procedures) were blinded. One of these studies reported blinding for both the participant and examiner (Antonio Barone et al., 2015) [16]; two studies reported the participant being blinded but not the examiner ((Ben Amara et al., 2021), (Nart et al., 2017)) [19,27]; and one study reported that the examiner was blinded after tooth extraction and the participant was not blinded (Stumbras et al., 2019) [30].

The details of the studies involved for the following section are outlined in Table 4. Blinding for statistical analysis was reported for eleven studies and histological analysis blinding was reported for ten studies. Calibration of histological measurements between examiners (inter-examiner) was reported in one out of the three studies which had more than one examiner. Out of the fifteen studies which had one examiner (intra-examiner), eleven didn’t report calibration of the histological measurements. In seventeen studies, the intervention was reported as identical. Three studies had incomplete outcome data and selective reporting, and seven studies reported a loss to follow-up.

All eighteen RCT’s had a parallel design in which eight of them, CONSORT (Consolidating Standards of Reporting Trials) was implemented. A split mouth design was conducted in four studies, three studies had a three-arm design, and one study had a four-arm design. Eight studies reported a source of funding, seven of these were companies which utilized their materials within the studies. Informed consent was undertaken in all the studies and ethical approval was gained in fifteen studies.

## 4. Discussion

The general classification of xenografts is based on their origin, predominantly being of either bovine or porcine origin. Two of the included studies involved Enamel Matrix Derivative which also has a porcine origin (Alkan et al., 2013) [15], (Mercado et al., 2021) [26]). Seldomly reported studies with an equine origin are also evident in the literature (De Cicco et al., 2020) [31], (Nishimura et al., 2020) [32]. The main findings of the research focused on the two main classifications of xenografts in relation to intra-classifications and we analysed within the bovine xenografts and inter-classifications i.e., between the bovine and porcine xenografts, as well as comparing xenografts to natural healing.

Each of the xenograft classifications and their findings was discussed with respect to their histomorphometric outcomes. The confounding factors was discussed, including a quality assessment of the studies in relation to their strength of evidence. From the literature, there is more evidence in relation to intra-classification in comparison to inter-classification of the xenografts. Only two of the studies directly compared bovine against porcine xenografts (Guarnieri, Stefano, et al., 2017) [22], (Lai et al., 2020) [25]). The mean N.B% for the bovine studies was 30.61% compared to 38.58% for the porcine studies and the mean R.G% for the bovine studies was 19.45% compared to 18.63% for the porcine group. The range in terms of the highest N.B% was MinerOss XP (porcine xenograft) with 57.43%, and the lowest is Bio-Oss collagen at 16.5%. The range in terms of the highest R.G% was Bio-Oss collagen and the lowest was Bio-Oss. This data seems to suggest that the porcine xenografts form more new bone, and porcine biomaterials resorb more than those of the bovine biomaterials [33]. However, it is hard to compare the studies as there are numerous confounding factors within them that make it difficult to draw robust conclusions, this will be elaborated upon in this discussion. Furthermore, clinical significance of these observed differences in NB and RG is unsubstantiated.

### Natural Healing 

A natural healing group was present in eight studies, five with bovine xenografts ((Ben Amara et al., 2021) [19], (Cardaropoli et al., 2012) [20], (Heberer et al., 2011) [24], (Santana et al., 2019) [28], (Stumbras et al., 2020) [30]) and three with porcine xenografts ((Antonio Barone et al., 2017) [18], (Crespi et al., 2011) [21], (Guarnieri, Testarelli, et al., 2017) [23]). The only xenograft out of these studies which had a higher bone formation than the natural healing group was a corticocancellous porcine xenograft (Crespi et al., 2011) [21]. This pattern is also corroborated with another systematic review involving five studies [24], hence further studies with natural healing groups are required to evaluate if the use of biomaterials in ARP is beneficial or a hindrance to new bone formation.

ARP is a treatment done prior to the placement of dental implants in most cases to provide more suitable bone, this is mirrored within these studies where the ARP procedures were done prior to the placement of implants in all 18 included studies. An international consensus [3] reports the benefit of ARP procedures and this systematic review supports this statement from a histomorphometric point of view. The ultimate goal of this research is to provide an insight into which xenograft protocols produce the best clinical results so that these can be used for future implant protocols. One of the studies looked into implant stability quotient subsequent to the placement of implants in grafted sites and showed that the sites which used EMD were superior compared to that of Bio-Oss [11]. The clinical relevance of this shows us that certain xenograft approaches may provide better osseointegration and hence, better implant related outcomes. Another study favoured the primary stability of implants in the bovine ARP sites compared to the porcine sites [9], this gives us an awareness into which xenografts provide better primary stability.

Furthermore, the healing times of the different studies can provide an understanding into which approaches produce outcomes that will be favourable for the best time to place implants. A flapped versus flapless study of ARP and subsequent implant placement showed successful outcomes for both approaches [12]. The clinical relevance of such a study can facilitate the best approach with respect to implant related outcomes. The study favoured the flapless approach in terms of more keratinized mucosa, better aesthetics and less recession illustrating the benefits on which approach to utilise.

One of the biggest reasons for the research is to find out how these different xenograft approaches will impact upon the success rates of the implants in the long term. Unfortunately, only one of the studies reported on implant survival rates [5], which had a favourable survival rate of Bio-Oss (100%) compared to Endobon (95%). However, to answer this question more accurately and reliably, future studies should have longer follow-up times and use a uniform criterion to measure success rates instead of survival rates, such of that proposed by Albrektsson [34], which used osseointegration when measuring success. Also, other parameters at the implant level, peri-implant soft tissue level, prosthetic level, and patient levels should be incorporated to provide a comprehensive outcome of which xenograft approach ultimately produces the best outcomes in terms of implant success [35].

The hypothesis to the research question that “there is an observational difference between the different xenograft biomaterials utilised in ARP in relation to their histomorphometric outcomes” can be considered as true as different outcomes of varying degrees for the biomaterials is clear within the studies. From the results, it is evident that the porcine xenografts had an overall higher mean new bone formation and a lower mean residual graft percentage, theoretically making them superior as a biomaterial. The hypothesis that “the various interventions of the ARP procedures have an influence on the histomorphometric outcomes” can also be considered as true as this is evident in the research in relation to numerous parameters i.e., flapped, or flapless approach and healing time. However, the extent of the observational difference between the biomaterials and how much of an influence the interventions have on the outcomes requires further investigation due to the confounding factors [35,36].

Firstly, some of the included studies had small sample sizes, larger sample sizes would provide more accurate data in terms of a smaller margin of error, identifying outliers and more accurate mean values. Multicentred trials will help in collecting larger samples of patients, however, interventions must be in place to help adherence to the study protocol to reduce the risk of deviation. Grafting in periodontally involved versus non periodontally involved as well as anterior posterior teeth would have different outcomes. Bungthong et al., aimed to assess the hard tissue changes around immediately placed implants in the posterior area using cone-beam computed tomography (CBCT) at a 6-month follow-up. After extracting teeth, twelve dental implants were inserted and filled with xenograft particles. CBCT images were captured right after the surgery and again at the 6-month mark. Analysis of the bone changes involved measuring vertical and horizontal bone thickness using ImageJ software. Statistical tests (paired t-test or Wilcoxon match-pair signed-rank test) were applied to assess changes in hard tissue values between immediate placement and 6-month follow-up. Independent t-tests or Mann–Whitney U tests were used to analyze dimensional changes on the buccal and lingual sides. The success rate was 100% for implant integration. Results at 6 months showed a decrease of 0.69 mm on the buccal side and 0.39 mm on the lingual side for vertical bone change. Horizontal bone thickness changes at various levels ranged from −0.22 mm to −0.70 mm. All measurement levels experienced a significant reduction in bone thickness within the 6-month period post-implant placement (*p* value < 0.05). This indicates that despite bone grafting, a decrease in bone thickness was observed after immediate implant placement. Hence, this technique could be considered as an alternative for posterior implant placement [37]. In light of the evolving landscape of bone grafting materials and techniques, it is worthwhile to acknowledge the potential role of equine bone grafts in future research endeavors. While our current study was designed to investigate specific grafting, modalities based on predefined criteria, the observation of equine bone grafts gaining prominence, particularly in regions such as the USA, raises intriguing questions about their applicability in different clinical contexts.

The eligibility criteria could also be improved in the studies regarding stricter observance of participants with ideally no smokers or a set number of cigarettes would help in standardization across the studies. A comprehensive list of health conditions and any thresholds deemed to be safe for implant treatment by liaising with the participants general medical practitioner for accurate medical histories would help remove any participants that may not be deemed eligible for the studies. A detailed periodontal assessment of candidates should be conducted and a uniform level of what is considered appropriate should be adhered to so that baseline characteristics are similar with consistent periodontal programmes for participants [38,39,40,41,42]. Standardizing the type of teeth being extracted, the reason for extraction and the socket defect after extraction is important so that all the sockets are similar in morphology. This will mean that the difficulty of the procedure is similar, and the biopsy will allow for a representative sample similar in nature. Furthermore, a clinician(s) of a consistent, high level of expertise throughout the studies would be best to provide a more uniform comparison of surgical intervention. The clinicians(s) should be uniformly calibrated and tested to provide a consistent level of treatment.

## 5. Conclusions

The current evidence included in this systematic review suggests that the two main xenograft biomaterials used in ARP procedures after tooth extraction, which are of bovine and porcine origin, displayed effectiveness in producing new bone. Nevertheless, there was difference between the different xenograft materials utilised for alveolar ridge preservation in relation to their histomorphometric parameters and that the various interventions of the alveolar ridge preservation procedures had an influence on the histomorphometric outcomes. There is some evidence which shows that porcine xenografts may yield a higher mean new bone formation percentage and a lower mean residual graft percentage. The studies looked at the tissue biopsy samples after 6 months post treatment to assess the new bone formation and showed that bovine xenografts, MinerOss X® had the highest new bone, and Bio-Oss® had the lowest residual graft percentage, making these the superior bovine xenografts relative to their histomorphometric outcomes. The porcine xenograft, MinerOss XP® had the highest new bone, and the lowest residual graft percentage. From the bovine xenografts, MinerOss X® had the highest new bone percentage whereas Bio-Oss® displayed the lowest residual graft percentage making these the superior bovine xenografts relative to their histomorphometric outcomes. It is also evident that an abundance of factors exists within the studies, broadly categorized into the parameters of population and pre-operative characteristics, intervention, and the histomorphometric analysis, which all have varying weight in affecting the histomorphometric outcomes. These findings indicate that both types of xenografts yield reliable results in terms of developing dental implant sites.

## 6. Future Work

The consideration of equine bone grafts could extend our understanding of grafting materials and broaden the spectrum of options available to clinicians. Future investigations might explore the efficacy, safety, and patient outcomes associated with equine bone grafts, taking into account factors such as regulatory approvals, regional preferences, and long-term success rates. By including equine bone grafts in the scope of future research, we can contribute to a more comprehensive evaluation of bone grafting strategies, catering to the diverse needs of patients and practitioners.

Future studies should ideally have the same healing time or have varying healing times of the same cohort of patients to provide a more accurate insight into what healing time produces the most bone and graft resorption. The studies had relatively short follow-up times, these should be longer to assess whether the ARP or subsequent implant placement within the grafted site has been successful long-term. The analysis of histomorphometry should also be standardized with respect to the processing of the biopsy, including the equipment and the method of analysing the slides. This should be done by an experienced examiner with adherence to clear outcome definitions to avoid confusing different tissue types. The correlation between the buccal bone thickness, tooth location and ARP have been studied although not in relation to their histomorphometric outcomes. This warrants further investigation as there could potentially be different methods of ARP utilized in different areas of the mouth or particular teeth which produce better results. Furthermore, there is evidence that the processing of xenografts by different manufacturers may produce different clinical responses which should also be explored to evaluate if their approaches differ. Additional studies comparing the physical characteristics, their handling and the quantity of biomaterials used within the extraction sites are justified to expand upon the limited research in this area.

## Figures and Tables

**Table 1:** Illustration of study characteristics in relation to their biomaterials, study design and number of patients. Key for table.

Author	Design/Setting/No. of Centres	Age (Years)-Mean (Range)	Gender	Number of Sockets	Groups (Test/Control), Materials	Confounding Factors
Alkan et al., 2013 [15]	RCT/Faculty of dentistry/Gazi University, Ankara, Turkey/1 centre	40–58 (range)	4 males, 6 females. 10 paients	20	T1-Enamel matrix derivative (*n* = 9). T2-Bio-Oss collagen (*n* = 9)	patients were none smokers, systemically healthy patients, Periodontal disease taken into consideration in elgibility criteria.
Barone et al., 2015 [16]	RCT/Versilia General Hospital (Dentistry Department), University of Pisa/1 centre	control-47, (35–71) Test-43.5, (21–67)	control (9 males, 8 females), test (5 males, 12 females). 34 patients	34	T, *n* = 17 flapless, secondary soft tissue healing C, *n* = 17 flap with primary closure-both groups recieved corticocancellous porcine bone and collagen membrane	smokers >10 cigarettes excluded, smokers <10 told to quit before and after surgery although compliance not monitored. Systemic health and periodontal disease taken into consideration in eligibility criteria.
Barone et al., 2013 [17]	RCT, 6 centres in Italy, Germany, Spain, university and private practice.	51 +/− 14	male-53%, female-47%. 38 patients	62	Test group-Endobon (bovine xenograft) *n* = 31 extraction sites, control group-Bio-Oss-bovine xenograft, *n* = 31 extraction sites	smokers ≥10 cigarettes excluded, systemic health taken into consideration in eligibility criteria. Periodontal disease not taken into consideration in elgibility criteria.
Barone et al., 2017 [18]	RCT, 5 centres- Universities of Pisa, Verona, Ancona (Italy), Murcia (spain) and a private practice	cort (48.2 +/− 12.8), coll (47.2 +/− 9.7), nat (46.9 +/− 10.8)	cort (males 14/females 16), coll (males 10/females 20), nat (males 12 /females 18). 90 patients	90	cortical porcine (cort (*n* = 30), collagenated corticocancellous porcine (coll (*n*= 30)), natural healing (*n* = 30)	smokers >10 a day excluded, systemic health taken into consideration in eligibility criteria. Periodontal disease not taken into consideration in elgibility criteria.
Ben Amara et al., 2021 [19]	RCT, 1 centre, Department of periodontology, Seoul National University, Korea	Test group (DBBM-C)-55.64, control (SH-49.42)	Test group (DBBM-C)-male/female-7/6, control (SH-male/female-5/8)-26 patients.	44	Test group (DBBM-C)-Bio-Oss collagen-*n* = 13, control (SH)-*n* = 13	smokers ≥20 excluded, systemic health taken into consideration in eligibility criteria. Periodontal disease taken into consideration in elgibility criteria.
Calasans-Maia, et al. 2014 [12]	RCT, 1 centre, (Dental Clinical Research Centre at Flu-minense Federal University-Rio de Janeiro), (Brazil)	44.55 (30–60)	T1 (Bio-Oss)-8 females:2 males, T2-(Osseus)-5 females:5 males. 20 patients	20	T1 (Bio-Oss) bovine xenograft-(*n* = 10) T2-(Osseus-bovine xenograft)-(*n* = 10)	smokers excluded, systemic health taken into conisderation in eligibility criteria. Periodontal disease not taken into consideration in elgibility criteria.
Cardaropoli et al., 2012 [20]	RCT, 1 centre, Private practice Torino, Italy.	47.2 +/− 12.9 (24–71)	17 females, 24 males. 41 patients.	48	Test-Bovine bone mineral (Bio-Oss) covered with a porcine collagen membrane (*n* = 24), Control-spontaneous healing (*n* = 24).	smokers >10 a day excluded, systemic health taken into consideration in eligibility criteria. Periodontal disease not taken into consideration in elgibility criteria.
Crespi et al., 2011 [21]	RCT, 1 centre, department of Dentistry, San Raffaele Hospital, Milan-Italy.	53.7 (32–70)	9 females, 6 males. 15 patients.	30	Test-corticocancellous porcine bone (Tecnoss) (*n* = 15), Control- natural healing (*n* = 15).	smokers excluded and systemic health taken into consideration in elgiibility criteria. Periodontal disease not taken into consideration in elgibility criteria.
Guarnieri, R., Stefano I, et al., 2017 [22]	RCT, setting/no of centres not reported	51.5 (35–63)	8 females, 10 males. 18 patients.	18	T1-MB-Bovine derived bone-MinerOss X (*n* = 10), T2-MP-Porcine dervied bone-MinerOss XP (*n* = 10).	smokers >10 a day excluded, systemic health taken into consideration in eligibility criteria. Periodontal disease not taken into consideration in elgibility criteria.
Guarnieri, R., Testarelli, L. et al. 2017 [23]	RCT, setting/no of centres not reported	46.7 (20–63)	14 males: 12 females. 26 patients.	26	G1-porcine derived bone + collagen membrane (*n* = 8), G2-Collagen membrane (*n* = 9), G3-Spontaneous healing (*n* = 9)	smokers >10 a day excluded, systemic health taken into consideration in eligibility criteria. Periodontal disease not taken into consideration in elgibility criteria.
Heberer et al., 2011 [24]	RCT, setting/no of centres not reported	49.9 (36–67)	10 females:15 males. 25 patients.	39	Test-Bovine derived bone-Bio-Oss collagen (20 extraction sites in 16 patients), Control-natural healing (19 extraction sites in 9 patients)	smokers excluded, systemic health taken into consideration in eligibility criteria. Periodontal disease not taken into consideration in elgibility criteria.
Lai et al., 2020 [25]	RCT, University setting 1 centre, Department of periodontics, UT health San Antonio School of Dentistry, San Antonio.	57 (24–82)	25 females:13 males. 38 patients	38	T1-Bovine derived bone-Bio-Oss (*n* = 21), T2-Porcine derived bone-Zcore (*n* = 17)	smokers >10 a day excluded, systemic health taken into consideration in eligibility criteria. Periodontal disease taken into consideration in elgibility criteria.
Mercado et al., 2021 [26]	RCT, 1 centre, private practice, NSW Australia.	52.5 +/− 10.8 years	69% female: 31% male. 42 patients	42	Test-Bio-Oss collagen (bovine) (*n* = 21) + Enamel matrix derivative (emdogain), Control-Bio-Oss-collagen	smokers excluded, systemic health taken into consideration in eligibility criteria.Periodontal disease taken into consideration in elgibility criteria.
Nart et al., 2017 [27]	RCT, 1 centre, Clínica Universitaria Odontológica in the Universidad Internacional de Cataluñna-Spain	56.76	not reported. 26 patients.	22	T1-DBBM-C (Bio-Oss collagen) plus a collagen membrane (*n* = 11), T2-DBBM (Bio-Oss) plus a collagen membrane (*n* = 11).	smokers >10 a day excluded, systemic health taken into consideration in eligibility criteria. Periodontal disease not taken into consideration in elgibility criteria.
Perelman-Karmon et al., 2012 [5]	RCT, no report of centre/setting	range (26–68)	16 females: 7 males. 23 patients.	23	Bio-Oss (bovine xenograft) with Bio-Gide (*n* = 12), Bio-Oss (bovine xenograft) alone (*n* = 11)	smokers excluded, systemic health taken into consideration in eligibility criteria. Periodontal disease not taken into consideration in elgibility criteria.
Santana et al., 2019 [28]	RCT, 1 centre, Goldman School of Dental Medicine, Boston University.	mean-42 +/− 8 years (SD)-range-34–52.	18 females: 14 males. 32 patients.	41	Allograft (*n* = 13), Bio-Oss (bovine xenograft) (*n* = 14), Blood coagulum (*n* = 14).	smokers excluded, systemic health taken into consideration in eligibility criteria. Periodontal disease not taken into consideration in elgibility criteria.
Sivolella et al., 2020 [29]	RCT, 1 centre (setting not reported (Italy)).	53.5	35% female: 65% male-20 patients	40	Endobon (bovine xenograft) (*n* = 20), Bio-Oss (bovine xenograft)-(*n* = 20).	smokers >10 a day excluded, systemic health taken into consideration in eligibility criteria. Periodontal disease taken into consideration in elgibility criteria.
Stumbras et al., 2020 [30]	RCT, 1 centre, Dept of Maxillofacial surgery, Lithuanian University of health sciences.	control group-51 +/− 14, G2-54 +/− 11	female (*n* = 26), male (*n* = 14). 40 patients.	40	G1 control-spontaneous healing (*n* = 10), G2-Bovine bone mineral-Bio-Oss and (resorbable native collagen membrane (*n* = 10), G3 freeze dried bone allograft + (*n* = 10), G4-Plasma Rich Growth Factors alone (*n* = 10)	smokers >10 a day excluded, systemic health taken into consideration in eligibility criteria. Periodontal disease taken into consideration in elgibility criteria.

**Table 2:** Illustration of the interventions in studies in relation to the aspects involved with tooth extraction and illustration of the interventions in studies in relation to the aspects involved with ARP.

Author	Groups (Test/Control), Materials	Clinician	Flap Raised/Releasing Incisions/Primary Closure	Reasons for Tooth Extraction	Atraumatic Extraction	Barrier Membrane	Pharmacological Treatment	Temporary Prosthesis Present	Healing Time before Measurement Biopsy and Monitoring during Healing	1. Number of Dropouts, 2. Adverse Events	Follow Up Time of Study	1. Number of Teeth + Type of Teeth/Socket Location, 2. Defect Morphology Reported in Eligibility Criteria
Alkan et al., 2013 [15]	T1-Enamel matrix derivative (*n* = 9). T2-Bio-Oss collagen (*n* = 9)	reported as 1 operator (EAA)-no mention of skill level	flapless, no releasing incisions, primary closure reported	not reported	yes	mucosal punch graft harvested from palate for primary coverage and then a free gingival graft 2–3 mm sutured to marginal gingivae	Post-op systemic antibiotics + 0.12% chlorhexidine mouthwash prescribed	not reported	3 months, review at 10 days and 4 weeks	1. one patient excluded due to necrotic grafts, 2. two grafts in one patient was necrotic	not reported	1. single rooted teeth in maxilla, 2. Socket defect taken into consideration in eligibility criteria (4 walled sockets included)
Barone et al., 2015 [16]	T, *n* = 17 flapless, secondary soft tissue healing C, *n* = 17 flap with primary closure-both groups received corticocancellous porcine bone and collagen membrane	1 surgeon (AB)	T-flapless, secondary soft tissue healing C-full thickness mucoperiosteal flap with primary closure, releasing incisions	included-fracture, decay, endodontic failure, perio disease	yes	collagen membrane-covered in control sites, exposed in test sites	antibiotic therapy-amoxicillin (2 g)/clindamycin (600 mg) 1 h pre-op and post-op (1 g amoxicillin/300 mg clindamycin, BD QDS). 0.2% CHX rinse 1 min pre-op rinse, and post-op-BD for 3 weeks. Post-op Naproxen sodium 550 mg BD as long as necessary	yes, not fitted sooner than 3 weeks post op and not permitted until adjusted	3 months, no monitoring	1. nil, 2. nil reported	5 years	1. maxillary premolars C (3), T (3), maxillary molars C (2), T (4), mandibular premolars C (2), T (1), mandibular molars C (10), T(9), 2. Socket defect taken into consideration in eligibility criteria
Barone et al., 2013 [17]	Test group-Endobon (bovine xenograft) n-31 extraction sites, control group-Bio-Oss-bovine xenograft, *n* = 31 extraction sites	reported as “operators”	flapless or flapped not reported, no releasing incisions, marginal closure reported	reported-severe decay, periodontal disease, tooth fracture, endodontic failure	yes	resorbable collagen membrane-Osseoguard (bovine derived)	not reported	not reported	6 months, review healing at 10 days (suture removal) and 2 weeks	1. nil, 2. site opening (t-39%, c-45%), membrane exposure (t-43%, c-42%), spillage/escape of graft material (t-41%, c-42%)	1 year	1. type of teeth-PM or M teeth (60% in maxilla), 2. socket defect reported + taken into consideration in eligibility criteria
Barone et al., 2017 [18]	cortical porcine (*n* = 30), collagenated corticocancellous porcine (n= 30), natural healing (*n* = 30)	reported as “clinicians who had received training”	flapless, no releasing incisions, membrane left exposed	reported-decay, endodontic failure, fracture	yes	collagen membrane (left exposed) for test groups (cort and coll), nil for control (nat)	antibiotic therapy-2 g amoxicillin (clindamycin 600 mg if allergic) 1 h pre op and post op-1 g amoxicillin (300 mg clindamycin if allergic) BD 5 days. Pre-op rinse with chlorhexidine mouthwash 0.2% for 1 min and BD for next 3 weeks.	not reported	3 months, no monitoring	not reported	5 years	1. premolar: molar ratio-cort (14:16), coll-10:20, nat (8:22), 2. socket defect reported but not in eligibility criteria
Ben Amara et al., 2021 [19]	Test group (DBBM-C)-Bio-Oss collagen-*n* = 13, control (spontaneous healing)-*n* = 13	reported as “clinicians”	flapless, no releasing incisions, no mention of primary closure	reported-periodontitis	yes	double layer of native collagen membrane	pre op rinse with 0.1% chlorhexidine for 1 min. medication (no mention of how long and when)-amoxicillin 500 mg tds, analgesics, chlorhexidine (0.1% mouthwash)	not reported	6 months, reviewed at day 10 (sutures removed)	1. reported (8), 2 not reported	6 months	1. test-max/mand-8/10, anterior/premolar/molar-2/2/14, control-max/mand-6/10, anterior/premolar/molar-0/1/15, 2. socket defect reported + in eligibility criteria
Calasans-Maia, et al. 2014 [12]	T1 (Bio-Oss) bovine xenograft-(*n* = 10) T2-(Osseus-bovine xenograft)-(*n* = 10)	1 surgeon	flapless extraction, releasing incisions post extraction with primary closure reported	reported-Periodontal, caries, tooth/root fracture	yes	not reported	pre op for ARP not reported. Only post op after implant therapy. 0.12% Chlorhexidine MW, BD-2 weeks post-op	not reported	6 months, healing was monitored 1, 7, 30, 90 days (sutures removed at day 14)	1. nil, 2. nil	12 months	1. no particular teeth for each test group-see Table 1 in journal, 2. Socket morphology not reported
Cardaropoli et al., 2012 [20]	Test-Bovine bone mineral (Bio-Oss) covered with a porcine collagen membrane (*n* = 24), Control-spontaneous healing (*n* = 24)	not reported	flapless, no releasing incision, no primary closure	reported-root fracture, periodontal issue, endodontic failure, caries	yes	porcine collagen	post op application of hyaluronic acid and amino acid gel TDS until wound closure. 1 g amoxicillin + clavulanate potassium every 12 h for 6 days + ibuprofen 600 mg evry 12 h for 3 days and 0.2% chlorhexidine gluconate every 8 h	told not to wear any prosthesis until healing was complete	4 months, weekly monitoring for 1st month post op. (suture removal at day 14 in control group)	1.nil, 2.nil	not reported	1. 4 first premolars, 12 s premolars, 32 molars-max/mand not reported, 2. socket morphology reported + in eligbility criteria.
Crespi et al., 2011 [21]	Test-corticocancellous porcine bone (Tecnoss) (*n* = 15), Control-natural healing (*n* = 15)	1 oral surgeon	not reported	not reported	not reported	collagen sheet	amoxicillin 1 g 1 h pre op +BD for 1 weeks post op	not reported	4 months, no report of monitoring	1. nil, 2. nil	not reported	1. premolar and molar regions max/mand-see Table 1 in journal, 2. Socket morphology considered in eligibility criteria
Guarnieri, R., Stefano I, et al., 2017 [22]	T1-MB-Bovine derived bone-MinerOss X (*n* = 10), T2-MP-Porcine derived bone-MinerOss XP (*n* = 10)	not reported	flapless, no releasing incisions, no primary closure	not reported	yes	porcine derived collagen resorbable membrane	2 g amoxicillin (clindamycin 600 mg if allergic) 1 h pre op and 0.2% chlorhexidine rinse 1 min pre op and 1 g amoxicillin (300 mg clindamycin if allergic) TDS 5 days post op with 0.2% chlorhexidine rinse BD for 3 weeks post op	not reported	4 months, reviewed at 3 months	1. reported, 2. nil	not reported	1. only report of premolar or molar tooth, 2. Socket defect not reported
Guarnieri, R., Testarelli, L. et al. [23]	G1-porcine derived bone +collagen membrane (*n* = 8), G2-Collagen membrane (*n* = 9), G3-Spontaneous healing (*n* = 9)	not reported	flapless, no releasing incisions, no primary closure	not reported	yes	G1 and G2 both collagen membrane, G3-nothing	2 g amoxicillin (clindamycin 600 mg if allergic) 1 h pre op and 0.2% chlorhexidine rinse 1 min pre op and 1 g amoxicillin (300 mg clindamycin if allergic) TDS 5 days post op with 0.2% chlorhexidine rinse BD for 3 weeks post op	not reported	4 months, reviewed at 3 months	1. reported, 2. nil	not reported	1. G1 (premolar/molar-4/4), G2 (premolar/molar-6/3), G3 (Premolar/molar-4/5), 2. Socket defect reported + taken into consideration in eligibility criteria
Heberer et al., 2011 [24]	Test-Bovine derived bone-Bio-Oss collagen (20 extraction sites in 16 patients), Control-natural healing (19 extraction sites in 9 patients)	reported as “clinician” for extraction, “surgeon” biopsy	flapless, no releasing incisions, no primary closure	not reported	yes	not reported	nil pre or post op	not reported	3 months, reviewed at day 1, 7, 60	1. nil, 2. nil reported	not reported	1. no particular teeth for each test group-see Table 1 and Table 2 in journal, 2. Socket defect reported + in eligibility criteria
Lai et al., 2020 [25]	T1-Bovine derived bone-Bio-Oss (*n* = 21), T2-Porcine derived bone-Zcore (*n* = 17)	12 surgeons (periodontal residents covered by board certified periodontal faculty)	flapped, no mention of releasing incisions, no primary closure	not reported	yes	dense polytetrafluoroethylene (d-PTFE) membrane	post op 500 mg amoxicillin evey 8 h for 7 days. (100 mg doxycycline every 12 h 7 days if allergic). Rinse 15 mL of 0.12% chlorhexidine BD for 30 s for 2 weeks	not reported	18–20 weeks. reviewed at 7–10 days (for suture removal) and 1 month post op for membrane removal	1. reported, 2. nil	not reported	1. not reported, 1 tooth per patient, 38 teeth in total, 2. Socket morphology reported + in eligibility criteria
Mercado et al., 2021 [26]	Test-Bio-Oss collagen (bovine) (*n* = 21) + Enamel matrix derivative (emdogain), Control-Bio-Oss- collagen	1 experienced periodontist (F.M)	flapless, no releiving incision, primary closure with free gingival graft	failed endodontic treatment (70%), tooth fracture/unrestorable, failed crown (30%)	yes	free gingival graft (anterior hard palate)	pre op for ARP-0.2% chlorhexidine MW 1 min pre op, 5–10 mg diazepam 1 h pre op. post op for ARP-not to brush site for 2 weeks, analgesics (paracetamol 500 mg and ibuprofen 150 mg) as needed. prescribed 0.12% chlorhexidine digluconate mouthwash mouthwash used BD, for 1st week, then surgical brush and chlorhexidine gel 0.12% application for 2nd and 3rd weeks. Same protocol for pre and post op for biopsy and implant surgery	not reported	4 months. review at 1, 3, 5 weeks and then 4 months	1. nil, 2. nil	not reported	1. Control-(central incisor (12), lateral incisor (7), canine (2). Test-central incisor (11), lateral incisor (8), canine (2), 2. Socket characteristics reported and taken into consideration in eligibility criteria
Nart et al., 2017 [27]	T1-DBBM-C (Bio-Oss collagen) plus a collagen membrane (*n* = 11), T2-DBBM (Bio-Oss) plus a collagen membrane (*n* = 11)	1 experienced faculty member of the periodontology department	flapped, no report of relieving incision, no primary closure	reported-not restorable due to lack of tooth structure	yes	A collagen membrane-Bio-Gide (porcine origin)	post op ARP-ibuprofen 600 mg every 8 h as needed and amoxicillin 500 mg every 8 h for 1 week, rinse with chlorhexidine 0.12% MW BD for 2 weeks. Post op implant surgery-same protocol as ARP	not reported	5 months, reviewed on weekly basis until soft tissue closure, then monthly until implant surgery	1. reported, 2. nil	not reported	1. maxilla-1st PM (7), 2nd PM (9), CI (1), Mandible-1st PM (3), 2nd PM (1), C (1), 2. Socket characteristics reported and taken into consideration in eligibility criteria
Perelman-Karmon et al., 2012 [5]	Bio-Oss (bovine xenograft) with Bio-Gide (*n* = 12), Bio-Oss (bovine xenograft) alone (*n* = 11)	not reported	flapped, relieving incisions as advancement flaps used, primary closure obtained via pedical flap and advancement flap for membrane group. Same for none membrane group although palatal area exposed for secondary healing	not reported	not reported	double layer of collagen membrane (Bio-Gide). (porcine origin)	post op Naproxen 275 mg, rinse with 0.2% chlorhexidine BD for 30 s for 2 weeks	not reported.	9 months, report of 1 month review and “frequently”	1. nil, 2. nil	not reported	1. Maxillary I (10), single root PM (8), mandibular single rooted PM (5), 2. Socket morphology taken into consideration in eligibility criteria
Santana et al., 2019 [28]	Allograft (*n* = 13), Bio-Oss (bovine xenograft) (*n* = 14), Blood coagulum (*n* = 14)	not reported	flapped if required, no report of releasing incisions, no primary closure	periodontal, endodontic or prosthetic reasons	yes	synthetic polymeric resorbable membrane (PEG)	wound cleansed with gauze and saline at suture removal (7 days), prescribed 0.12% chlorhexidine MW to use BD. Informed not to brush surgical area	reported-adjusted and free from surgical site	6 months, review at 7 days (and suture removal)	1. reported, 2. nil	not reported	1. I, C, PM teeth-see Table 1 in journal, 2. Socket morphology not taken into consideration
Sivolella et al., 2020 [29]	Endobon (bovine xenograft) (*n* = 20), Bio-Oss (bovine xenograft)-(*n* = 20)	reported as one operator (SS)	flapped, releasing incisions reported, primary closure reported	not reported	yes	collagen membrane (bovine derived)-OsseoGuard	1 g amoxicillin tablet every 12 h for 6 days (500 mg clarithromycin BD if allergic), on morning of surgery. Appropriate analgesics (not mentioned)	not reported.	4 months, 10 days review (and suture removal)	1. reported, 2. nil	24 months	1. PM and M sites,-Maxilla (Endobon 70%, Bio-Oss (70%). Mandible Endobon (30%), Bio-Oss (30%), 2. Socket morphology taken into consideration in eligibility criteria
Stumbras et al., 2020 [30]	G1 control-spontaneous healing (*n* = 10), G2-Bovine bone mineral-Bio-Oss and resorbable native collagen membrane (*n* = 10), G3 freeze dried bone allograft + (*n* = 10), G4-Plasma Rich Growth Factors alone (*n* = 10)	1 surgeon (A.S)	flapless, no releasing incisions, no primary closure reported	endodontic cause, fracture, periodontal cause, caries	yes	G2, G3-both resorbable native collagen membrane-Jason membrane (porcine origin)	post op rinse BD with 0.12% chlorhexidine MW for 2 weeks	reported-not to wear for 2–3 weeks post op and adjusted	3 months, review at 14 days (and suture removal)	1. reported, 2. nil	12 months	1. maxillary single rooted anterior teeth only, 2. Socket morphology taken into consideration in eligibility criteria

**Table 3:** Illustration of characteristics of histomorphometric analysis.

Author (Year)	Biomaterials Groups	Measure of Dispersion	New Bone %	Residual Graft %	Histomorphometric Outcomes e.g % Bone/% Residual Graft) and Definitions
1. Alkan et al., 2013 [15]	EMD	Mean +/− SD	34.57 +/− 25.67	-	
	Bio-Oss Collagen-Bovine	Mean +/− SD	28.80 +/− 16.14	-	newly formed bone (no clear definition)
2. Barone et al., 2015 [16]	flapless (Test)-corticocancellous porcine bone (MP3) and collagen membrane	Mean +/− SD	22.5 +/− 4.3	18.2 +/− 5.2	newly formed bone, residual grafted material, marrow spaces (no clear definition although explanation in histological analysis)
		Median	21	19	
	Flapped (Control)-corticocancellous porcine bone (MP3) and collagen membrane	Mean +/− SD	22.5 +/− 3.9	18.2 +/− 6.1	
		Median	21	18	
3. Barone et al., 2013 [17]	Endobon-Bovine (Test)	Mean +/− SD	28.5% +/− 20	-	newly formed bone, residual xenograft (not reported). No clear definition although explanation in histological analysis
	Bio-Oss-Bovine (control)	Mean +/− SD	31.4% +/− 18.1	-	
4. Barone et al., 2017 [18]	cortical porcine (Apatos)	Mean +/− SD	36.8 +/− 19.1	15.5 +/− 8.4	Newly formed bone %, Residual graft particles %, None mineralsied tissue %.no report of definition
	corticocancellous porcine (MP3)	Mean +/− SD	41.4 +/− 20.6	14.9 +/− 7.3	
	natural healing	Mean +/− SD	44.0 +/− 14.7	-	
5. Ben Amara et al., 2021 [19]	depoteinised bovine bone mineral with 10% collagen (Bio-Oss Collagen)	Mean +/− SD	30.87 +/− 17.27	14.08 +/− 10.01	proportion of bone %, remnant graft %, Connective tissue %. No clear definition although explanation in histological analysis.
	natural healing	Mean +/− SD	53.94+/16.52	-	
6. Calasans-Maia, et al. 2014 [12]	T1 Osseous, bovine xenograft	Mean +/− SD	-	10.6 +/− 16.2	mean newly formed vital bone area fraction (%), mean newly formed connective tissue (%), mean remaining Biomaterial (%). No clear definition although explanation in histological analysis.
	T2 Bio-Oss, Bovine xenograft	Mean +/− SD	-	22.5 +/− 7.9	
7. Cardaropoli et al., 2012 [20]	Test-Bovine bone mineral (Bio-Oss) covered with a porcine collagen membrane	Mean +/− SD	26.34 +/− 16.91	18.46 +/− 11.18	new bone %, residual graft %, mineralised fraction %, C.T + bone marrow %. Defintions explained.
	Control-spontaneous healing	Mean +/− SD	43.82 +/− 12.23	-	
8. Crespi et al., 2011 [21]	test-corticocancellous porcine bone (Tecnoss)	Mean +/− SD	-	34.4 +/− 5.1	New bone % (amount of mineralsied and vasularised tissue as % of total tissue volume), connective tissue %, Residual graft %. No clear definition although explanation in histological analysis
	control-natural healing	Mean +/− SD	-		
9. Guarnieri, R., Stefano I et al., 2017 [22]	T1-MB-Bovine dervied bone-MinerOss X	Mean +/− SD	49.08 +/− 3.7	13.49 +/− 2.8	Newly formed bone %, connective tissue %, Residual graft %, Osteoid tissue. No definitions reported.
	T2-MP-Porcine dervied bone-(MinerOss XP)	Mean +/− SD	57.13 +/− 2.8	11.74 +/− 4.7	
10. Guarnieri, R., Testarelli, L et al., 2017 [23]	G1-porcine derived bone +collagen membrane (MinerOss XP)	Mean (SD)	57.43 (4.8)	16.57 (3.8)	%bone/tissue area, %C.T/tissue area, % graft/tissue area. No clear definition although explanation in histological analysis.
	G2-Collagen membrane	Mean (SD)	60.01 (3.2)	-	
	G3-Spontaneous healing	Mean (SD)	48.85 (2.37)	-	
11. Heberer et al., 2011 [24]	Test-Bovine dervied bone-Bio-Oss Collagen	Mean (range)	25% (4–23%)	17% (4–37%) in molar region, 13% (4–31% in premolar region).	mean new bone formation %, mean mean Graft remnant %, Mean Connective tissue %. Definition of viable bone reported, explanation in histological analysis reported.
	Control-natural healing	Mean (range)	44% (3–79%)	-	
12. Lai et al., 2020 [25]	T1-Bovine dervied bone-Bio-Oss	Mean (SD)	-	20.47 (15.29)	Vital bone % (presence of osteocytes in mineralised tissue), residual graft % (absence of osteocytes in mineralsed tissue), Connective tissue/other % (remaining tissue). Defintions reported
	T2-Cancellous Porcine dervied bone-Z core	Mean (SD)	-	19.52 (9.19)	
13. Mercado et al., 2021 [26]	Test-Bio-Oss Collagen (bovine) + Enamel matrix derivative (emdogain)	Mean +/− SD	45.1 +/− 8.8	20.3 +/− 7.2	New bone % (lighter stained mineralised tissue with osteocytes in lacunae), residual graft % (mineralised tissue with empty lacunae). Soft tissue and marrow spaces % (remaining area fraction not marked as new bone/residual graft). Definitions reported.
	Control Bio-Oss collagen	Mean +/− SD	16.5 +/− 6.9	36.8 +/− 8.8	
14. Nart et al., 2017 [27]	T1-DBBM-C (Bio-Oss Collagen) plus a collagen membrane,	Mean	37.68 +/− 13.38	16.00 +/− 11.60	Volume of newly formed bone %, graft particles % and connective tissue %. No clear definition although explanation in histological analysis.
	T2-DBBM (Bio-Oss) plus a collagen membrane.	Mean	33.44 +/− 17.82	13.14 +/− 8.32	
15. Perelman-Karmon et al., 2012 [5]	Bio-Oss (bovine xenograft) with Bio-Gide	Mean +/− SD	40.8 +/− 10.61 one area fraction %.	-	mean Bone Area Fraction % at apical, middle and crestal regions of the root. Definition reported.
	Bio-Oss (bovine xenograft) alone	Mean +/− SD	29.7 +/− 7.21 Bone area fraction %	-	
16. Santana et al., 2019 [28]	Allograft + PEG	Mean	n/a	n/a	mineralised bone %, graft particles %. No definitions reported.
	Bio-Oss (bovine xenograft) + PEG	Mean	28.18	8.89	
	Blood coagulum + PEG	Mean	47.81	-	
17. Sivolella et al., 2020 [29]	Endobon (Bovine xenograft)	Mean +/− SD	33.4 +/− 19.9	21.3 +/− 15.4	new bone formation %, residual bone graft %, Marrow or fibrous tissue %, new bone formation that is vital %. Definitions reported.
	Bio-Oss (bovine xenograft)	Mean +/− SD	32.4 +/− 20.4	15.8 +/− 14.5	
18. Stumbras et al., 2020 [30]	G1-spontaneous healing	Mean +/− SD	-	n/a	newly formed mineralised tissue %, Newly formed none mineralsied tissue %, residual graft %. No definitions reported.
	G2-bovine bone mineral Bio-Oss with resorbable native collagen membrane	Mean +/− SD	-	45 +/− 19	
	G3-freeze dried bone allograft with resorbable native collagen membrane	-	n/a	n/a	
	G4-Plasma Rich in Growth Factors (PRGF) alone.	-	n/a	n/a	

**Table 4:** Illustration of the quality assessment of the included studies.

Author (Year)	Eligibility Criteria Defined	Representative Population Group	Random Sequence Generation	Allocation Concealment	Blinding of Participant and Examiner (Surgeon)	1. Blinded for Statistical Analysis 2. Blinded for Histological Analysis	Calibration: 1. Intra-Examiner. 2. Inter-Examiner
Alkan et al., 2013 [15]	yes	yes, split mouth, although small sample size	yes, coin toss	not reported	not reported for both	1. not reported, 2. yes	2. inter-examiner (2 examiners)-no calibration reported
Barone et al., 2015 [16]	yes, clearly	yes	yes, computerised and envelope method	not reported	yes both participant and examiner	1. not reported, 2. not reported	1. intra-examiner (1 clinician-(GI))-no calibration reported
Barone et al., 2013 [17]	yes	yes, split mouth so baseline variable similar	yes, opaque cards used	not reported	not reported for both	1. not reported, 2. not reported	2. not calibrated and not reported between 6 centres
Barone et al., 2017 [18]	yes, clearly	yes	yes, computersied and envelope method	not reported	not reported for both	1. yes, 2. not reported	2. yes calibration reported
Ben Amara et al., 2021 [19]	yes, clearly	yes	yes, computersied and envelope method.	not reported	blinding of participant but not examiner	1. yes, 2. not reported	1. yes calibration reported
Calasans-Maia, et al. 2014 [12]	yes, clearly	yes	yes, envelope system method	not reported	not reported for both	1. not reported, 2. yes	1. not reported
Cardaropoli et al. 2012 [20]	yes	yes	yes, computerised	not reported	not reported for both	1. not reported, 2. yes	1. not reported
Crespi et al., 2011 [21]	yes	yes, split mouth so baseline variable similar	yes, no method stated	not reported	not reported for both	1. not reported, 2. not reported	1. not reported
Guarnieri, R., Stefano I. et al., 2017 [22]	yes, clearly	yes	yes, computerised and envelope method	not reported	not reported for both	1 yes, 2. not reported	1. not reported
Guarnieri, R., Testarelli, L. et al. 2017 [23]	yes, clearly	yes	yes, computerised and envelope method	not reported	not reported for both	1. yes, 2. yes	1. not reported
Heberer et al., 2011 [24]	yes	yes	yes, computerised	not reported	not reported for both	1. yes, 2. yes	1. yes calibration reported
Lai et al., 2020 [25]	yes, clearly	yes	yes, envelope method	not reported	not reported for both	1. not reported, 2. yes	1. yes calibration reported
Mercado et al., 2021 [26]	yes, clearly	yes	yes, envelope method	not reported	not reported for both	1. not reported, 2. yes	1. only cbct measurements calibrated, not histology
Nart et al., 2017 [27]	yes, clearly	yes	yes, computerised	yes	participant blinded, examiner not reported	1. yes, 2. yes	1. yes calibration reported
Perelman-Karmon et al., 2012 [5]	yes	no	yes, coin toss	not reported	not reported for both	1. not reported, 2. yes	1. not reported
Santana et al., 2019 [28]	yes	yes	yes, no method reported	not reported	not reported for both	1. not reported, 2. not reported	1. not reported
Sivolella et al., 2020 [29]	yes	yes	yes, blinded card method	not reported	not reported for both	1. not reported, 2. not reported	1. not reported
Stumbras et al., 2020 [30]	yes, clearly	yes	yes, computerised	not reported	participant not blinded, examiner blinded after tooth extraction	1. not reported, 2. yes	1. not reported
